# Tissue-Specific Trade-Offs Between Biomineralisation and Antioxidant Responses in *Magallana gigas* Infected with Boring Sponges *Pione vastifica*

**DOI:** 10.3390/antiox15050596

**Published:** 2026-05-08

**Authors:** Ekaterina Kladchenko, Ekaterina Vodiasova, Olga Gostyukhina, Daria Lavrichenko, Viktoria Uppe, Elina Chelebieva

**Affiliations:** A.O. Kovalevsky Institute of Biology of the Southern Seas of RAS, 119991 Moscow, Russia

**Keywords:** *Magallana gigas*, boring sponges, *Pione vastifica*, oxidative stress, biomineralisation, gene expression, shell repair, energy trade-off, flow cytometry

## Abstract

Infestation by boring sponges poses a serious problem for Pacific oyster *Magallana gigas* (Thunberg, 1793) aquaculture. This study aimed to assess the effect of *Pione vastifica* sponge infestation on the oysters’ capacity for shell repair, antioxidant defence status, and hemocyte functional state. We analysed the expression of *VEGF* pathway genes and biomineralisation enzymes, molecular chaperones (*Hsp70*, *Hsp90*), growth arrest and DNA damage gene (*Gadd45α*), antioxidant enzyme activity and lipid peroxidation levels in the hemolymph and various mantle parts (central and outer-edge). Intracellular reactive oxygen species (ROS) levels and mitochondrial membrane potential in hemocytes were evaluated. The results showed that infection significantly increases intracellular ROS levels in hemocytes without changing mitochondrial membrane potential. Oxidative damage was localised primarily in the central mantle contacting the damaged shell. In the outer-edge mantle responsible for shell growth, marked upregulation of *SodMn*, *Cat*, and *Gadd45α* was observed, coupled with suppression of *VEGF-R* receptor expression and organic matrix genes. Heat shock protein expression decreased in all examined tissues of infected molluscs. Our results demonstrate that shell damage induced by boring sponges triggers a tissue-specific reorganisation of physiological priorities, manifesting as a bioenergetic trade-off where limited energy resources are reallocated from the ATP-demanding process of biomineralisation to sustain antioxidant defence and cell survival.

## 1. Introduction

The growing global demand for protein sources has driven the expansion of aquaculture, with bivalve molluscs representing one of its most valuable sectors [1]. The Pacific oyster (*Magallana gigas*) occupies a leading position in terms of cultivation volume worldwide [2]. Currently, Pacific oysters are cultivated on all continents except Antarctica [1]. The popularity of this species is attributed to its rapid growth rates, tolerance to variations in abiotic factors, and favourable nutritional profile [3,4,5]. However, in recent years, global climate change has facilitated the range expansion of various pathogens into new regions and increased the frequency of diseases among bivalve molluscs [6]. Specifically, mariculture facilities have reported increased Pacific oyster mortality linked to two major pathogens: OsHV-1 µVar (causing POMS with up to 100% spat mortality) and *Vibrio aestuarianus* subsp. *francensis* (causing up to 30% mortality in market-size oysters) [7,8,9]. Given that oysters are predominantly consumed raw, this increased disease frequency limits aquaculture development due to potential food safety concerns.

In addition to viral, parasitic, and bacterial infections, various epibionts settling on mollusc shells can negatively affect the functional state of the organism [10]. Among these epibionts are boring sponges of the family Clionaidae. These sponges settle on bivalve shells and excavate networks of tunnels. Since the bivalve shell serves as a barrier protection, the ability to continuously maintain regeneration in response to constant mechanical damage is crucial for the successful defence of Pacific oysters against predatory and various ecological threats [11]. However, such a host response requires the reallocation of energy resources from basal metabolism to the restoration of the protective barrier integrity. Consequently, molluscs may exhibit reduced filtration activity and growth rates, compromised non-specific immune response, elevated oxidative stress, and increased mortality if the balance shifts in favour of the pathogen [10,12,13,14,15].

Disruption of shell integrity and the organism’s barrier function induces histological changes in tissues, increases the risk of opportunistic microflora invasion, and affects the composition of the mollusc-associated microbiome [16,17]. In oysters infected with boring sponges, reduced microbial diversity is observed in the gills, and the total number of microorganisms on the shells of such individuals exceeds thresholds typical for healthy individuals [17,18]. Damage and subsequent microbial load initiate the activation of the molluscan immune response. Hemocytes, as effectors of bivalve immunity, migrate to damaged zones, participating in shell repair and inflammatory response [19,20]. We have previously shown that infestation by boring sponges induces changes in the cellular composition of *M. gigas* hemolymph, as well as increased phagocytic activity coupled with enhanced production of reactive oxygen species (ROS) [15]. Despite the protective role of ROS in pathogen elimination, their excessive generation under chronic stress conditions can deplete antioxidant system reserves and lead to oxidative cellular damage. However, the molecular mechanisms regulating shell repair processes and antioxidant status in different mantle regions of oysters infested with boring sponges remain poorly understood.

Our research focused on evaluating how boring sponges influence the shell repair ability of *M. gigas*, along with their antioxidant defence. It was hypothesized that epibiont pests compromise the functional state and key protective systems of oysters with damaged shells. To test this hypothesis, we assessed the expression of genes involved in the vascular endothelial growth factor (*VEGF*) pathway and those encoding biomineralisation-related enzymes. We also measured the expression of molecular chaperones (*heat shock proteins 70* and *90*), which play a crucial role in protecting cells against heat and other stresses, as well as growth arrest and DNA damage (*Gadd45α*). Furthermore, we hypothesised that the nature of the stress response would differ spatially: while oxidative damage would primarily occur in the central mantle (in direct contact with the damaged shell), the outer-edge mantle (responsible for growth) would mount a distinct, pre-emptive defensive response to preserve tissue viability, even at the cost of growth processes. To test this, we compared antioxidant enzyme activity and thiobarbituric acid reactive substance (TBARS) levels in the central and outer-edge mantle of *M. gigas*. This study provides important insights into the antioxidant defences and biomineralisation capacity of ecologically and economically important oysters infected with boring sponges, contributing to a mechanistic understanding of host–pathogen interactions and the bioenergetic costs of biofouling.

## 2. Materials and Methods

### 2.1. Animal Collection and Maintenance

**Figure 1:**
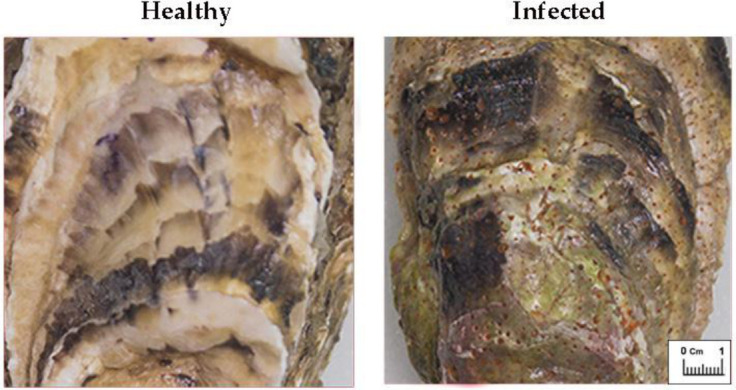
Shell morphology of infested with boring sponges and healthy *Magallana gigas*.

Twenty Pacific oysters (age 5 years, weight 90.7 ± 7.1 g, shell length 17.2 ± 2.1 cm) were sourced from Mariculture Ltd. (Sevastopol Bay, Black Sea). Infected oyster shells exhibited characteristic red bioerosion patterns, with a frequency of 31.5% ± 12.5% of the total shell area. Healthy control oysters were selected from the same mariculture facility based on an absence of visible bioerosion patterns, shell discolouration or sponge excavation channels, as verified by a Nexcope NSZ608T stereomicroscope (Nexcope, Ningbo Yongxin Optical Co., Ltd., Ningbo, China). This ensured that the control and infected groups were exposed to identical environmental conditions prior to experimenting (Figure 1). Specimens were delivered to the laboratory in dry transport containers and placed into an aquarium prepared with artificial seawater Red Sea Salt, (Red Sea Fish Pharm Ltd., Eilat, Israel). The animals were acclimated for a period of 14 days in tanks with recirculating seawater and aerated with ambient air (oxygen content: 8 mg L^−1^, temperature of 15 ± 1 °C and salinity of 18 ± 1). The seawater in the tank was replaced on a daily basis. During the acclimation process, oysters were provided with a daily diet consisting of a mixture of microalgae species, including *Tetraselmis viridis* and *Chlorella vulgaris* f. *suboblonga*, at a biomass ratio of 1:1. Algae blend was added to the experimental tanks to ensure the maintenance of a density of approximately 4 × 10^5^ cells mL^−1^. Both the infected and control groups were fed identically.

### 2.2. Hemolymph and Tissue Collection

The hemolymph was collected from the adductor muscle using a sterile 2 mL syringe fitted with a 21-gauge needle. Prior to aspiration, the syringe was pre-filled with 0.5 mL of ice-cold artificial seawater (ASW) adjusted to a salinity of 18. This procedure was implemented to prevent the aggregation of the hemocytes. Immediate analysis was carried out for hemocytes used in functional assessments, including intracellular ROS production and mitochondrial membrane potential evaluation. For all remaining analyses, hemocyte and soft tissue specimens were snap-frozen in liquid nitrogen and preserved at −80 °C for a maximum period of 3 months until further processing.

### 2.3. Mitochondrial Membrane Potential and Intracellular ROS Accumulation

Hemocyte parameters were evaluated via flow cytometry using a MACSQuant Analyzer (Miltenyi Biotec GmbH, Bergisch Gladbach, Germany). To exclude bacterial contamination and cellular debris, an FSC threshold was applied, and 10,000 events were recorded per sample. Target hemocyte populations were isolated using forward and side scatter (FSC/SSC) gating, and raw data were processed with MACSQuantify software (version 3.0.2). Changes in mitochondrial membrane potential (MMP) were assessed using a rhodamine-based fluorescence assay [21]. Rhodamine 123 (Rh123; Sigma-Aldrich, St. Louis, MO, USA), a lipophilic cationic dye that selectively accumulates in active mitochondria, is routinely employed for MMP evaluation [22]. Intracellular ROS levels in hemocytes were quantified using DCF-DA (Sigma-Aldrich, St. Louis, MO, USA) according to a previously described method [12]. Briefly, cell suspensions were incubated with 10 μM DCF-DA at 5 °C in the dark for 45 min, followed by fluorescence measurement (Ex 485 nm/Em 528 nm).

### 2.4. Biochemical Assay of Enzymatic Activity

Biochemical analyses of mantle tissues were performed following previously established protocols [12,15]. Briefly, tissues stored at −80 °C were thawed on ice and homogenized in cold 20 mM Tris-HCl buffer (pH 7.5) with 0.5 mM EDTA. Supernatants were collected after centrifugation (11,000× *g*, 20 min, 4 °C) [23] and used for enzyme assays. Catalase (CAT) activity was measured at 405 nm based on the H_2_O_2_–ammonium molybdate reaction [24] and expressed as μmol H_2_O_2_ min^−1^ mg protein^−1^. Superoxide dismutase (SOD) activity was determined using the NBT assay of Nishikimi et al. [25] at 540 nm (arbitrary units). All enzyme measurements were performed in triplicate at 25.0 ± 0.5 °C [26]. Protein concentration was quantified by using the Lowry method [27] at 750 nm, and lipid peroxidation was assessed as TBARSs (MDA equivalents) at 532 nm following Ohkawa et al. [28].

### 2.5. RNA Expression Analysis

Total RNA was isolated from *M. gigas* mantle tissue using the RNeasy Mini Kit (Qiagen, Hilden, Germany) following the manufacturer’s protocol. Following the exposure trial, ten mantle samples per treatment were harvested. Extracted RNA was treated with DNase I (15 min at 37 °C) and resuspended in 50 µL of nuclease-free water. RNA integrity was verified by 1.5% agarose electrophoresis with ethidium bromide staining, while concentration was quantified using a Qubit 4.0 fluorometer (Thermo Fisher Scientific, Waltham, MA, USA). Subsequently, cDNA was synthesised using the MMLV kit (Evrogen, Moscow, Russia), following the manufacturer’s protocol. The reverse transcription process was conducted in a final volume of 15 μL, comprising 500 ng of purified RNA. The primer sequences utilised in this study are enumerated in Table 1 [29,30,31,32]. The study targeted stress response transcripts *(Hsp70, Hsp90, Gadd45), antioxidant *enzymes *(Cat, SodMn, SodCu/Zn), and biomineralisation-related genes (Cas-kin1, Cas-kin2, VEGF, and VEGF receptor). β-actin *and *ef1α *were selected as reference genes. Quantitative real-time PCR analysis was performed on a LightCycler 96 real-time PCR instrument (Roche Diagnostics, Mannheim Germany) using a qPCRmix-HS kit with SYBR Green I dye (Evrogen, Russia). The reaction mixture (total volume 25 µL) contained 1 µL of cDNA, 0.5 µM of each primer, and Milli-Q water. Thermal cycling consisted of an initial denaturation at 95 °C for 30 s, followed by 40 cycles of 95 °C for 5 s, 55 °C for 20 s, and 72 °C for 10 s, with a final extension at 72 °C for 7 min. A melting curve analysis (55–95 °C, ramp rate 0.5 °C/s) was included to verify amplicon specificity. All samples were run in technical triplicate alongside no-template controls. Quantification cycle (Cq) values were determined using Roche LightCycler software (v.1.1). Amplification efficiencies were validated via standard curves generated from serial cDNA dilutions. The stability of reference genes was evaluated using BestKeeper v.1 [33]. Relative gene expression was calculated using the ∆Cq method [34], normalized to the geometric mean of β-actin and ef1α.

### 2.6. Statistical Analyses

Data were assessed for compliance with parametric test assumptions: normality was evaluated via the Shapiro–Wilk test, while homogeneity of variances was examined using Levene’s test. Due to the limited number of biological replicates (*n* = 10 per group), we adopted a bootstrap approach to evaluate group differences. This method provides robust inference under small-sample conditions without requiring distributional assumptions [35,36]. Two biologically relevant comparisons were performed for each biomarker:

1. Infected vs. healthy individuals within the same tissue (e.g., hemolymph or mantle).
2. Differences between tissues (e.g., hemolymph vs. mantle1) within the same health status (healthy or infected).

For each comparison, we estimated the mean difference and its 95% confidence interval (CI) using 2000 bootstrap resamples with replacement. A difference was considered statistically meaningful if the 95% CI excluded zero, a criterion that avoids reliance on *p*-values while maintaining interpretability in terms of effect direction and precision. All analyses were implemented in RStudio (v4.4.0).

## 3. Results

### 3.1. Hemocyte Functional Response to Infection

**Figure 2:**
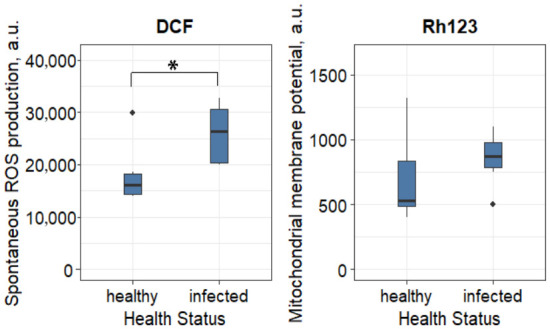
Functional status of hemocytes in *Magallana gigas* infested with boring sponges. Statistical significance was quantified by 95% bootstrap confidence interval. Differences are significant at *p* < 0.05 (*).

Infection with *P. vastifica* significantly increased intracellular ROS production in hemocytes of *M. gigas* (diff = 7759.1 a.u., 95% CI: 1733.4–13,300.4), whereas the MMP remained unchanged (Figure 2).

### 3.2. Antioxidant Capacity

**Figure 3:**
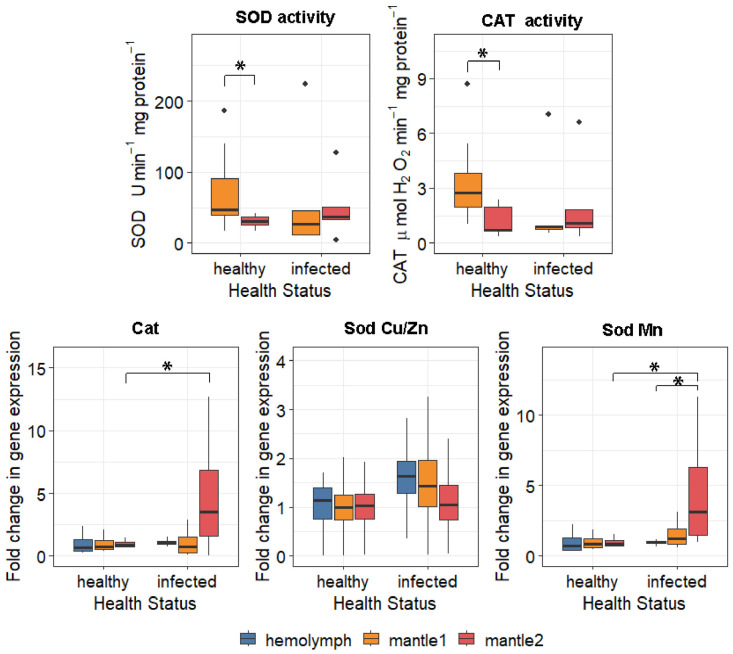
Antioxidant status and oxidative damage in the mantle of Pacific oysters *M. gigas*, infected with boring sponges. Statistical significance was quantified by the 95% bootstrap confidence interval. Differences are significant at *p* < 0.05 (*).

In healthy oysters, activities of CAT and SOD were significantly lower in the outer-edge mantle (mantle2) compared to the central mantle (mantle1) (Table S2), while expression of corresponding genes (*Cat*, *SodMn*, *SodCu*/*Zn*) did not differ between tissues (Figure 3). Enzyme activities and gene expression levels became comparable between mantle regions in infected individuals. Notably, in infected individuals, *SodMn* expression and SOD activity were upregulated in mantle2 relative to mantle1 (diff = 3.66, 95% CI: 0.27–7.97).

### 3.3. Oxidative Damage and Stress Response

**Figure 4:**
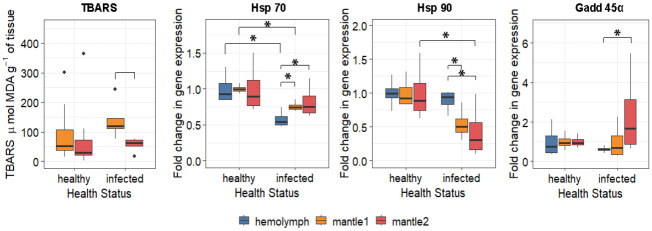
Profile of oxidative stress (TBARS) and expression of stress-related proteins (*Hsp70*, *Hsp90*) and genotoxicity marker (*Gadd45α*) in tissues of *M. gigas* infected with boring sponge. Statistical significance was quantified by 95% bootstrap confidence interval. Differences are significant at *p* < 0.05 (*).

TBARSs were significantly elevated in mantle1 of infected oysters (diff = −83.3, 95% CI: −139.9 to −35.8) (Figure 4). Concurrently, expression of the stress response gene *Gadd45α* was upregulated in mantle2 of infected individuals (diff = 1.74, 95% CI: 0.15–3.76), while *Hsp70* expression decreased in both the hemolymph (diff = −0.42, 95% CI: −0.64 to −0.23) and mantle1 (diff = −0.24, 95% CI: −0.32 to −0.17). *Hsp90* expression showed tissue-specific downregulation: it was reduced in mantle1 (diff = −0.46, 95% CI: −0.74 to −0.18) and mantle2 (diff = −0.57, 95% CI: −1.05 to −0.08) but unchanged in the hemolymph. While infection did not significantly alter *Gadd45α* expression compared to healthy controls within individual tissues (Table S1), a significant difference emerged between tissues within the infected group: expression was higher in the outer-edge mantle (mantle2) compared to the central mantle (diff = 1.74, 95% CI: 0.15–3.76; Table S2).

### 3.4. Biomineralisation-Related Gene Expression

**Figure 5:**
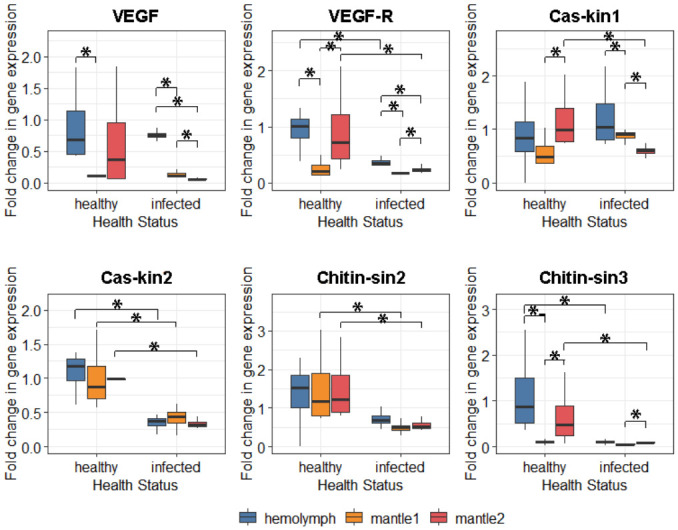
VEGF pathway and biomineralisation gene expression enzymes in tissues of *M. gigas*. Statistical significance was quantified by 95% bootstrap confidence interval. Differences are significant at *p* < 0.05 (*).

Infection induced widespread suppression of genes involved in shell formation and repair (Figure 5). Expression of the *VEGF* pathway receptor (*VEGF-R*) was significantly downregulated in the hemolymph (diff = −0.57, 95% CI: −0.87 to −0.20) and mantle2 (diff = −0.69, 95% CI: −1.49 to −0.11; Table S1), although *VEGF* ligand expression remained unchanged. *Casein kinase* and *chitin synthase* isoforms showed differential tissue-specific suppression. *Cas-kin2* expression was consistently reduced across all tissues examined (hemolymph, mantle1, and mantle2; Table S1), whereas *Cas-kin1* was downregulated only in the outer-edge mantle (mantle2) (diff = −0.59, 95% CI: −1.13 to −0.16). Similarly, chitin synthase genes were suppressed in specific compartments: *Chitin-sin2* expression decreased in both mantle regions (mantle1 and mantle2), while *Chitin-sin3* was downregulated in the hemolymph and outer-edge mantle (mantle2) (Table S1).

## 4. Discussion

Our study demonstrates that infestation by the boring sponge *P. vastifica* induces a significant increase in intracellular ROS levels in the hemocytes of the Pacific oyster *M. gigas*. These findings align with our previous observations on the immune response to bioerosion [12,13]. While bivalves naturally generate ROS during basal metabolism and in response to exogenous stressors [37,38,39], the mechanism driving this oxidative burst under chronic sponge infestation differs distinctly from a typical mitochondrial hypermetabolic reaction. Typically, stress-induced ROS production is linked to intensified cellular respiration, as mitochondria are major sources of superoxide alongside ATP synthesis [40,41]. However, in our work, the MMP remained unchanged between infected and control groups. This stability suggests that the observed oxidative load was not driven by a general upregulation of mitochondrial respiration. Instead, the primary source of radicals likely stems from non-mitochondrial systems, specifically the activation of NADPH oxidases in hemocytes as part of an immune “respiratory burst” triggered by tissue damage and microbial invasion [42,43]. Alternatively, the preservation of the MMP amidst high ROS levels may indicate protective uncoupling of oxidative phosphorylation—a mechanism that reduces proton back-pressure on the respiratory chain, thereby limiting hydrogen peroxide production [44,45]. Although elevated ROS levels are traditionally viewed as cytotoxic metabolic byproducts [46], emerging evidence highlights their dual role as signalling molecules in regeneration [47]. ROS are known to play a central role in activating signal cascades during the early phases of bone repair in higher vertebrates [48]. Furthermore, inhibiting ROS formation has been shown to block tissue regeneration in Platynereis, Nematostella, and *Danio rerio* (Hamilton, 1822) [47,49]. While the elevated ROS in hemocytes likely reflects an NADPH oxidase-dependent ‘respiratory burst’ aimed at pathogen clearance and immune surveillance [42,43], the moderate ROS increase observed in specific mantle zones may serve a distinct, conserved signalling function to initiate tissue repair and biomineralisation processes [47,49,50]. However, it is crucial to distinguish between this potential signalling role and overt oxidative damage. We propose that while low-to-moderate ROS concentrations may act as regenerative signals in the growth zone, excessive radical accumulation in the contact zone (central mantle) exceeds the antioxidant system’s buffering capacity, resulting in cellular damage rather than productive signalling.

Cellular protection against oxidative damage relies on antioxidant defence systems [51,52]. In our study, shell damage coincided with oxidative stress in the mollusc mantle. These data corroborate previous findings on the physiological impacts of shell damage and other stressors in bivalves. We previously reported increased lipid peroxidation in *M. gigas* infected with boring sponges [13,14]. Similarly, infection of Pacific oysters by the polychaete *Polydora* sp. led to increased *SOD Cu/Zn* expression in the heart, likely in response to heightened oxidative load [53]. Comparable patterns of lipid peroxidation were observed in *Ruditapes decussatus* (Linnaeus, 1758) and *Perna perna* (Linnaeus, 1758) mussels exposed to acidification, another shell-damaging factor [54,55]. However, most studies analyse whole tissues, overlooking local variations. The tissue-specific response of the antioxidant system revealed in our study underscores a strategic allocation of defence resources. In healthy oysters, lower SOD and CAT activities in mantle2 compared to mantle1 reflect a baseline state with minimal oxidative pressure in the growth zone. Upon infection, a pronounced trade-off emerged: while lipid peroxidation surged in the central mantle (indicating localized damage at the shell interface), the outer-edge mantle exhibited induction of antioxidant defences. Expression of *Cat*, *SodMn*, and *Gadd45α* increased exclusively in the outer mantle. Such discrepancies in Cat and SodMn expression and enzyme activity suggest that expression activation serves as an early preparatory signal, whereas achieving full functional enzyme capacity may require additional time, cofactor mobilisation or metabolic resources. Furthermore, spatial segregation suggests prioritized protection of the mantle edge—the tissue responsible for shell accretion and linear growth [30,56]. The upregulation of *Gadd45α*, a gene involved in cell cycle arrest, DNA repair, apoptosis regulation, and the induction of antioxidant genes [57], alongside antioxidant enzymes in the outer zone, likely represents a protective mechanism to halt proliferation and prevent DNA damage propagation in this critical growth area. This aligns with reports of reduced growth rates in sponge-infested oysters [18], indicating that the organism actively suppresses growth to ensure cell survival under stress.

Beyond antioxidant defence, resistance to environmental stressors can be enhanced via heat shock proteins [58,59]. These proteins facilitate the removal of irreversibly damaged proteins, shielding cells from abnormal polypeptides [60]. In our study, *Hsp* expression levels did not differ between tissues in uninfected oysters, unlike in infected individuals. *Hsp70* expression decreased significantly in hemocytes and the mantle region contacting the damaged shell, whereas *Hsp90* expression declined only in the outer mantle of infected mollusks. In bivalve research, heat shock proteins serve as markers of stress levels and overall functional status [58,59]. Based on the existing literature regarding *Hsp* functions in invertebrates and vertebrates [61,62], we infer analogous roles here. Specifically, *Hsp90* binds pro-apoptotic factors to prevent apoptosis, while *Hsp70* can directly enhance the activity of transcription factors mediating antioxidant defence [63,64]. Consequently, in the outer mantle, preserved chaperone activity combined with *Gadd45α* induction and antioxidant enzyme upregulation ensures protection of the shell growth zone. Conversely, the simultaneous decline in *Hsp70* and *Hsp90* in the central mantle and hemocytes, coupled with a lack of antioxidant compensation, indicates functional exhaustion and heightened vulnerability to oxidative stress in these tissues.

Shell damage by the boring sponge led to widespread suppression of genes governing shell formation and repair, particularly in the hemolymph and outer-edge mantle. The significant downregulation of the *VEGF* pathway receptor (*VEGF-R*) in circulating hemocytes and the growth zone implies a blockade of the signalling cascade required for mobilizing resources for shell construction [65,66]. Moreover, the suppression of organic matrix genes (*Casein kinase* and *Chitin synthase* isoforms) in the outer mantle reinforces this conclusion. *Chitin synthases* and *chitinases* regulate the formation and degradation of chitin fibres, respectively—the foundation of the organic shell framework [67]. However, *Chitin-sin2* function extends beyond biomineralisation: in shrimp, it modulates hemocyte apoptosis in innate immunity [68], and in mammals, it participates in cell survival and proliferation [69]. Given the functional role of the outer mantle, reduced *Chitin-sin2* expression in this zone of infected oysters likely correlates with halted shell growth. According to the Cellular Energy Allocation concept [70], when energy demands for maintenance and defence (e.g., antioxidant synthesis, immune response, DNA repair) exceed supply, organisms must reallocate limited reserves away from costly functions like growth and reproduction. The observed suppression of biomineralisation genes in the outer mantle—precisely where antioxidant defence is maximal—strongly supports the hypothesis that *M. gigas* sacrifices shell growth to finance the high metabolic cost of surviving chronic oxidative stress. Interestingly, the expression of specific biomineralisation genes (*Cas-kin1*, *Chitin-sin3*) in the central mantle remained comparable to controls despite high oxidative damage. This local retention may reflect an attempt to maintain repair processes directly at the bioerosion site. However, without upregulation above control levels—and given severe oxidative damage and hemocyte dysfunction—the efficacy of such local repair is likely limited. Although bivalves possess a remarkable capacity for rapid shell repair following acute injury [71,72], the chronic nature of continuous sponge bioerosion appears to exceed the organism’s regenerative potential, forcing a shift from “growth” to “survival” mode.

## 5. Conclusions

This study demonstrates that *P. vastifica* infestation forces *M. gigas* to shift its energy budget from shell growth to stress defence, disrupting physiological homeostasis. While the central mantle suffers oxidative damage and immune exhaustion, the outer-edge mantle activates a robust pre-emptive antioxidant response (upregulated *SOD*, *CAT*, *Gadd45α*) to protect the growth zone. Concurrent downregulation of key biomineralisation genes (*VEGF-R*, casein kinase, chitin synthase) confirms a compensatory trade-off, explaining the reduced growth rates and structural vulnerability observed in infected oysters. Although limited local repair attempts occur in the central mantle, they are insufficient against continuous bioerosion. Future research should quantify the energetic fluxes of this trade-off and determine whether biomineralisation suppression is reversible, which is critical for managing recovery in affected aquaculture populations.

## Figures and Tables

**Table 1:** Primer sequence of target and reference genes for RT-qPCR.

EST Name	Forward 5′–3′	Reverse 5′–3′	GenBank Accession Number
*Sod Mn*	ACAAAGTCAATCAGTGCCCT	CCATTGCCTCTGCCAGT	EU420128
*Sod Cu/Zn*	CCAGAGGATCACGAGAGGC	GCGTTTCCGGTCGTCTT	AJ496219
*Cat*	TTCGTCATATCGGGTTTACTTCTG	CCTTGTCACGTCCTGCCATT	AM853618
*β-actin*	AGTTGGTGACGATGCCGTGTTC	CAGAGCTGTGTTTCCCTCCATTGT	AF026063
*Hsp70*	AACGGTATCCTGAATGTGTC	CTTCTCGTCTTCCTGCTTG	AF144646
*Hsp90*	CGAGGAAGCAGAAGCAGAG	ATGTCACCAGACGGTTAGATAC	AF144646
*Gadd45a*	TGCTGGAGAACAAGTCGGAC	TCAAAGAAGTCGGCCAGCAT	XM_011446511
*Ef1α*	AGTCACCAAGGCTGCACAGAAAG	TCCGACGTATTTCTTTGCGATGT	AB122066.1
*VEGF*	CCGGTGCATGTGTACCAATA	TGATTTCCTCGTCAGTCATTCC	XM_011451443.2, LOC105343926
*VEGF-R*	CAAATGCACCTTGACCCAATAC	CGGTCTATGGCTCTGCATAAA	XM_011457891.1, LOC105348465
*Cas-kin1*	GGAGGTGGCTGTTAAGTTAGAG	GCGAGCAGAAGTTGAAGAGA	XM_011448074.2, LOC105341513
*Cas-kin2*	CGATGAAGCAGAGATCCCATTA	CAAACAGCACATGACCAACTAC	XM_011419091.2, LOC105320946
*Chitin-sin2*	CGCAACAATGGGCAATAGAG	CTGATATCGAGGCGGTGAATAG	XM_011425423.2, LOC105325734
*Chitin-sin3*	GTACAAATGGGCTCTGGGAT	GTCGAACTCACACTGGAAGAA	JH816899.1, CGI_10012656

## Data Availability

The original contributions presented in this study are included in the article/Supplementary Materials. Further inquiries can be directed to the corresponding author.

## Supplementary Materials

The following supporting information can be downloaded at: https://www.mdpi.com/article/10.3390/antiox15050596/s1, Table S1: Bootstrap estimates of mean differences and 95% confidence intervals for infection-induced effects on molecular, cellular, and biochemical markers in bivalve *M. gigas*; Table S2: Bootstrap estimates of mean differences and 95% confidence intervals for tissue-specific effects on molecular, cellular, and biochemical markers in bivalve *M. gigas*.

## Abbreviations

The following abbreviations are used in this manuscript:

A.U.	Arbitrary unit
CAT	Catalase
Cq	Threshold cycle value
DCFH-DA	2′,7′-dichlorodihydrofluorescein diacetate
DNA	Deoxyribonucleic acid
EF1	Elongation factor-1α
FDA	Fluorescein Diacetate
GAL	Galectin
LEC	Lectin
PCR	Polymerase chain reaction
VEGF	vascular endothelial growth factor
ROS	Reactive oxygen species
RT-qPCR	Quantitative real-time PCR
SOD	Superoxide dismutase
TBARS	Thiobarbituric acid reactive substance
MMP	mitochondrial membrane potential
EDTA	ethylenediaminetetraacetic acid
NBT	nitro blue tetrazolium
MDA	malondialdehyde
