# Supplementary material for: Tissue-Specific Trade-Offs Between Biomineralisation and Antioxidant Responses in Magallana gigas Infected with Boring Sponges Pione vastifica
Source: Antioxidants (Basel). 2026 May 8;15(5):596. doi: 10.3390/antiox15050596 (PMC13203924; doi:10.3390/antiox15050596)
Supplement: Supplementary file 1 [file antioxidants-15-00596-s001.zip › antioxidants-4280968-supplementary.pdf]

Table S1. Bootstrap estimates of mean differences and 95% confidence intervals for infection-induced effects on molecular, cellular, and biochemical markers in the bivalve *M. gigas.*

**Tissue**	**Marker**	**diff**	**lower**	**upper**	**significant**
mantle1	CAT	-1.439	-4.012	1.375	FALSE
mantle1	SOD	-9.883	-78.532	77.482	FALSE
mantle1	TBARS	43.911	-41.264	122.422	FALSE
mantle2	CAT	0.953	-0.579	3.320	FALSE
mantle2	SOD	20.488	-9.658	62.344	FALSE
mantle2	TBARS	-22.693	-114.375	39.229	FALSE
**hemolymph**	**DCF**	**7759.092**	**1733.364**	**13300.390**	**TRUE**
hemolymph	Rh123	179.134	-79.381	395.884	FALSE
hemolymph	*Cas-kin1*	0.358	-0.481	1.225	FALSE
**hemolymph**	***Cas-kin2***	**-0.741**	**-1.001**	**-0.428**	**TRUE**
hemolymph	*Cat*	0.090	-0.807	0.856	FALSE
hemolymph	*Gadd45 a*	-0.383	-1.150	0.205	FALSE
**hemolymph**	***Hsp70***	**-0.423**	**-0.636**	**-0.233**	**TRUE**
hemolymph	*Hsp90*	-0.111	-0.337	0.117	FALSE
hemolymph	*Sod CuZn*	0.630	-0.397	1.665	FALSE
hemolymph	*Sod Mg*	-0.036	-0.853	0.599	FALSE

hemolymph	*VEGP*	-0.139	-0.747	0.326	FALSE
**hemolymph**	***VEGP-R***	**-0.566**	**-0.873**	**-0.200**	**TRUE**
hemolymph	*Chitin-sin2*	-0.629	-1.385	0.299	FALSE
**hemolymph**	***Chitin-sin3***	**-1.066**	**-1.967**	**-0.357**	**TRUE**
mantle1	*Cas-kin1*	0.294	-0.021	0.546	FALSE
**mantle1**	***Cas-kin2***	**-0.593**	**-1.083**	**-0.202**	**TRUE**
mantle1	*Cat*	0.099	-1.063	1.411	FALSE
mantle1	*Gadd45 a*	-0.040	-0.791	0.855	FALSE
**mantle1**	***Hsp70***	**-0.244**	**-0.321**	**-0.168**	**TRUE**
**mantle1**	***Hsp90***	**-0.455**	**-0.735**	**-0.176**	**TRUE**
mantle1	*Sod CuZn*	0.558	-0.724	1.849	FALSE
mantle1	*Sod Mg*	0.575	-0.437	1.701	FALSE
mantle1	*VEGP*	0.022	-0.019	0.072	FALSE
mantle1	*VEGP-R*	-0.091	-0.259	0.035	FALSE
**mantle1**	***Chitin-sin2***	**-1.028**	**-2.034**	**-0.245**	**TRUE**
mantle1	*Chitin-sin3*	-0.045	-0.100	0.005	FALSE
**mantle2**	***Cas-kin1***	**-0.588**	**-1.132**	**-0.162**	**TRUE**
**mantle2**	***Cas-kin2***	**-0.653**	**-0.708**	**-0.585**	**TRUE**
**mantle2**	***Cat***	**3.942**	**0.074**	**8.980**	**TRUE**
mantle2	*Gadd45 a*	1.335	-0.247	3.460	FALSE

mantle2	*Hsp70*	-0.180	-0.543	0.149	FALSE
**mantle2**	***Hsp90***	**-0.569**	**-1.054**	**-0.080**	**TRUE**
mantle2	*Sod CuZn*	0.154	-0.892	1.231	FALSE
**mantle2**	***Sod Mg***	**3.657**	**0.303**	**8.067**	**TRUE**
mantle2	*VEGP*	-0.600	-1.342	0.001	FALSE
**mantle2**	***VEGP-R***	**-0.693**	**-1.487**	**-0.108**	**TRUE**
**mantle2**	***Chitin-sin2***	**-0.965**	**-1.816**	**-0.285**	**TRUE**
**mantle2**	***Chitin-sin3***	**-0.583**	**-1.209**	**-0.085**	**TRUE**

Table S2. Bootstrap estimates of mean differences and 95% confidence intervals for tissue-specific effects on molecular, cellular, and biochemical markers in the bivalve *M. gigas.*

Type	Marker	diff	lower	upper	significant
**healthy**	**CAT**	**-2.278**	**-4.041**	**-0.840**	**TRUE**
**healthy**	**SOD**	**-43.412**	**-84.700**	**-7.651**	**TRUE**
healthy	TBARS	-14.564	-106.427	92.591	FALSE
infected	CAT	0.113	-2.697	3.365	FALSE
infected	SOD	-11.994	-103.467	57.403	FALSE
**infected**	**TBARS**	**-83.335**	**-139.996**	**-35.764**	**TRUE**
healthy	*Cas-kin1*	-0.309	-1.047	0.363	FALSE
healthy	*Cas-kin1*	0.299	-0.511	1.069	FALSE
**healthy**	***Cas-kin1***	**0.593**	**0.074**	**1.162**	**TRUE**
healthy	*Cas-kin2*	-0.079	-0.546	0.431	FALSE
healthy	*Cas-kin2*	-0.094	-0.351	0.216	FALSE
healthy	*Cas-kin2*	-0.011	-0.437	0.340	FALSE
healthy	*Cat*	0.014	-1.023	1.026	FALSE
healthy	*Cat*	-0.003	-0.890	0.795	FALSE
healthy	*Cat*	0.003	-0.742	0.627	FALSE
healthy	*Gadd45 a*	-0.001	-0.764	0.683	FALSE
healthy	*Gadd45 a*	0.004	-0.755	0.625	FALSE
healthy	*Gadd45 a*	0.003	-0.424	0.418	FALSE

healthy	*Hsp70*	0.003	-0.209	0.164	FALSE
healthy	*Hsp70*	-0.002	-0.329	0.358	FALSE
healthy	*Hsp70*	-0.002	-0.253	0.319	FALSE
healthy	*Hsp90*	-0.001	-0.252	0.265	FALSE
healthy	*Hsp90*	-0.004	-0.377	0.414	FALSE
healthy	*Hsp90*	-0.001	-0.374	0.413	FALSE
healthy	*Sod CuZn*	0.009	-0.893	0.937	FALSE
healthy	*Sod CuZn*	0.006	-0.890	0.897	FALSE
healthy	*Sod CuZn*	0.013	-0.972	0.962	FALSE
healthy	*Sod Mg*	-0.003	-0.970	0.871	FALSE
healthy	*Sod Mg*	0.008	-0.846	0.734	FALSE
healthy	*Sod Mg*	-0.005	-0.621	0.569	FALSE
**healthy**	***VEGP***	**-0.789**	**-1.398**	**-0.334**	**TRUE**
healthy	*VEGP*	-0.239	-1.097	0.668	FALSE
healthy	*VEGP*	0.556	-0.051	1.292	FALSE
**healthy**	***VEGP-R***	**-0.677**	**-1.026**	**-0.302**	**TRUE**
healthy	*VEGP-R*	-0.004	-0.694	0.814	FALSE
**healthy**	***VEGP-R***	**0.680**	**0.072**	**1.443**	**TRUE**
healthy	*Chitin-sin2*	0.187	-0.963	1.494	FALSE
healthy	*Chitin-sin2*	0.184	-0.892	1.447	FALSE

healthy	*Chitin-sin2*	0.001	-1.200	1.162	FALSE
**healthy**	***Chitin-sin3***	**-1.075**	**-1.979**	**-0.374**	**TRUE**
healthy	*Chitin-sin3*	-0.500	-1.580	0.468	FALSE
**healthy**	***Chitin-sin3***	**0.575**	**0.087**	**1.246**	**TRUE**
infected	*Cas-kin1*	-0.367	-0.992	0.106	FALSE
**infected**	***Cas-kin1***	**-0.662**	**-1.304**	**-0.178**	**TRUE**
**infected**	***Cas-kin1***	**-0.281**	**-0.415**	**-0.130**	**TRUE**
infected	*Cas-kin2*	0.069	-0.134	0.252	FALSE
infected	*Cas-kin2*	-0.010	-0.122	0.116	FALSE
infected	*Cas-kin2*	-0.074	-0.239	0.113	FALSE
infected	*Cat*	0.006	-0.933	1.251	FALSE
infected	*Cat*	3.805	-0.114	9.225	FALSE
infected	*Cat*	3.778	-0.423	9.132	FALSE
infected	*Gadd45 a*	0.359	-0.296	1.207	FALSE
**infected**	***Gadd45 a***	**1.743**	**0.153**	**3.764**	**TRUE**
infected	*Gadd45 a*	1.410	-0.339	3.597	FALSE
**infected**	***Hsp70***	**0.181**	**0.059**	**0.289**	**TRUE**
**infected**	***Hsp70***	**0.244**	**0.045**	**0.486**	**TRUE**
infected	*Hsp70*	0.063	-0.118	0.284	FALSE
**infected**	***Hsp90***	**-0.349**	**-0.576**	**-0.087**	**TRUE**
**infected**	***Hsp90***	**-0.473**	**-0.793**	**-0.092**	**TRUE**

infected	*Hsp90*	-0.119	-0.475	0.281	FALSE
infected	*Sod CuZn*	-0.064	-1.497	1.302	FALSE
infected	*Sod CuZn*	-0.467	-1.673	0.755	FALSE
infected	*Sod CuZn*	-0.392	-1.743	0.998	FALSE
infected	*Sod Mg*	0.585	-0.240	1.603	FALSE
**infected**	***Sod Mg***	**3.660**	**0.271**	**7.968**	**TRUE**
infected	*Sod Mg*	3.073	-0.414	7.746	FALSE
**infected**	***VEGP***	**-0.624**	**-0.705**	**-0.542**	**TRUE**
**infected**	***VEGP***	**-0.699**	**-0.773**	**-0.626**	**TRUE**
**infected**	***VEGP***	**-0.073**	**-0.127**	**-0.033**	**TRUE**
**infected**	***VEGP-R***	**-0.197**	**-0.263**	**-0.146**	**TRUE**
**infected**	***VEGP-R***	**-0.135**	**-0.222**	**-0.051**	**TRUE**
**infected**	***VEGP-R***	**0.062**	**0.010**	**0.122**	**TRUE**
infected	*Chitin-sin2*	-0.220	-0.493	0.029	FALSE
infected	*Chitin-sin2*	-0.151	-0.383	0.079	FALSE
infected	*Chitin-sin2*	0.071	-0.136	0.288	FALSE
infected	*Chitin-sin3*	-0.047	-0.095	0.004	FALSE
infected	*Chitin-sin3*	-0.013	-0.061	0.041	FALSE
**infected**	***Chitin-sin3***	**0.036**	**0.015**	**0.055**	**TRUE**
